# Mast Cells Are Critical for Protection against Peptic Ulcers Induced by the NSAID Piroxicam

**DOI:** 10.1371/journal.pone.0023669

**Published:** 2011-08-12

**Authors:** Daniel D. Hampton, Laura P. Hale

**Affiliations:** 1 Division of Gastroenterology, Department of Medicine, Duke University Medical Center, Durham, North Carolina, United States of America; 2 Department of Pathology, Duke University Medical Center, Durham, North Carolina, United States of America; 3 The Human Vaccine Institute, Duke University Medical Center, Durham, North Carolina, United States of America; McGill University, Canada

## Abstract

Many commonly used non-steroidal anti-inflammatory drugs (NSAIDs) also cause gastrointestinal toxicity, including the development of life-threatening peptic ulcers. We report that mast cell-deficient mice have an extremely high incidence of severe peptic ulceration when exposed to the NSAID piroxicam. This enhanced ulcer susceptibility can be reversed by reconstitution with mast cells. Furthermore, wild type mice treated with diphenhydramine hydrochloride, a commonly used antihistamine that blocks histamine H1 receptors, develop a similarly high incidence of peptic ulcers following piroxicam exposure. The protective effect of mast cells is independent of TNF, blockade of H2 receptors, or acid secretion. These data indicate a critical role for mast cells and the histamine that they produce in prevention and/or repair of piroxicam-induced gastric mucosal injury. Additional studies will be required to determine whether this represents a NSAID class effect that can be exploited to develop novel therapeutic strategies to limit the incidence of NSAID-induced side effects in humans.

## Introduction

Peptic ulcers result from an imbalance between factors that damage the mucosa and the normal mucosal defense and repair mechanisms. Examples of factors that predispose to ulceration include *Helicobacter pylori* infection, stomach acid, stress, alcohol, tobacco, and non-steroidal anti-inflammatory drugs (NSAIDs) [1], [2]. Thirty million people use NSAIDs daily, with frequent use reported by up to 70% of some populations [3]–[6]. This widespread use makes NSAID-associated peptic ulcer disease (PUD) a significant public health concern which is associated with high annual treatment costs [7], [8].

Inhibition of cyclooxygenases (COX) that produce prostaglandins (PGs) is thought to account for both the anti-inflammatory effects and the gastrointestinal (GI) toxicities of NSAIDs. PGs coordinate secretion of protective mucus, surfactant, and bicarbonate, reduce acid secretion, decrease epithelial permeability, increase mucosal blood flow, and enhance inflammation [9], [10]. Although it was hypothesized that selective inhibition of COX-2 could control pain while preventing adverse effects, COX-2-specific NSAIDs still have GI toxicity [11], [12] and they also increase risk of cardiovascular events [13]–[15]. Studies in COX-1 knockout mice suggest that NSAID-induced damage can be unrelated to COX-1 inhibition and PGE_2_ levels [16], [17]. Possible mechanisms include bile reflux, since gastric ulcers related to systemic NSAID administration can be reduced or eliminated by blocking or diverting bile flow [18], [19]. NSAIDs may also compromise mucosa through their inherent acidic properties or through effects on epithelial cell proliferation and apoptosis, mucosal blood flow, or production of growth factors or antioxidants.

Ulcer healing requires formation of granulation tissue, angiogenesis, re-epithelialization, proliferation, and tissue remodeling. Mast cells have been shown to contribute to wound healing in the skin by modifying neutrophil trafficking, angiogenesis, and remodeling of the extracellular matrix. In this study, wild type (WT) and mast cell-deficient mice were used to investigate the role of mast cells in NSAID-induced gastrointestinal injury and healing. Results showed that mast cell-deficient mice were exquisitely sensitive to peptic ulceration induced by the NSAID piroxicam. This sensitivity could be reversed by mast cell reconstitution or by administration of antihistamines to WT mice. Taken together, the data suggest a critical role for mast cells and the histamine they produce in repair of piroxicam-induced gastric mucosal injury.

## Results

### Mast cell-deficient *Sash* mice are highly susceptible to piroxicam-induced peptic ulceration

**Figure 1:**
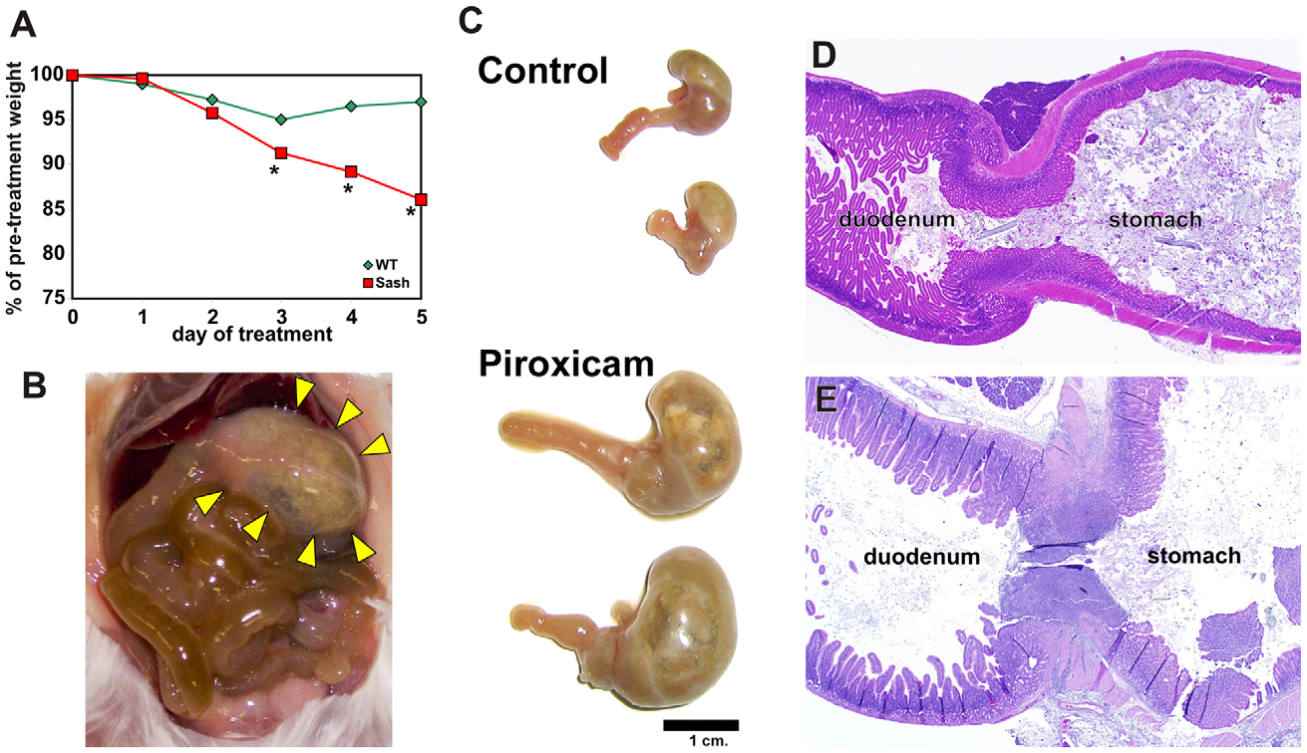
Mast cell-deficient *Sash* mice are highly susceptible to NSAID-induced peptic ulcers. A. Mean weight data is shown for 13 *Sash* and 10 wild type (WT) mice exposed to 200 ppm piroxicam in their diet. SEMs ranged from 0.4–1.6% of pre-treatment weight; error bars were omitted for clarity. The rapid weight loss correlates with decreased intake of food and water. Euthanasia for humane reasons was typically required on days 4–6. * indicates p≤0.05 relative to WT mice. Piroxicam-exposed *Sash* mice demonstrated marked gastric distension by gas and fluid (B, bottom half of panel C) compared with *Sash* mice not exposed to piroxicam (labeled “control” in panel C). Histologic examination showed ulceration at the gastroduodenal junction (E) of *Sash* mice, with marked inflammatory cell infiltration and edema that led to gastric outlet obstruction. WT mice (D) do not typically develop ulcers or changes in stomach size with piroxicam exposure.

**Figure 2:**
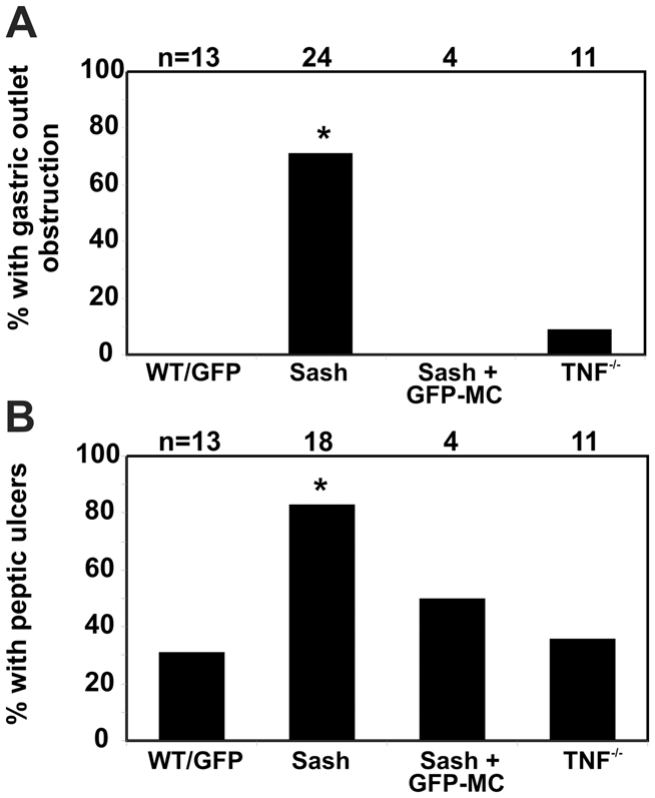
Mast cell-deficient mice are more susceptible to peptic ulceration when exposed to piroxicam. Significantly more *Sash* mice developed grossly evident gastric outlet obstruction defined as gastric enlargement >4× normal (A) and histologically detected peptic ulcers (B) when exposed to piroxicam, compared with WT or *Tnf* ^−/−^ mice. Both the increased sensitivity to peptic ulcers and gastric outlet obstruction appeared to be mast cell-dependent, since they reversed when *Sash* mice were reconstituted with mast cells (Sash+GFP-MC), but were not TNF-dependent since *Tnf* ^−/−^ mice had a similar incidence of gastric ulceration and gastric outlet obstruction as WT mice. * indicates p≤0.05 relative to WT.

Mast cell-deficient *Sash* mice were noted to lose weight very rapidly when fed powdered chow containing 200 ppm piroxicam (Figure 1A). Necropsy showed marked gastric distension, with stomachs increased to ≥4× normal size in 17 of 24 (71%) of exposed mice (Figure 1B, C; Figure 2A). Retained food, fluid, and air suggested gastric outlet obstruction. Ulcers were present at the gastro-duodenal junction in 15 of 18 *Sash* mice examined histologically (Figure 1D; Figure 2B), with marked inflammatory cell infiltration and edema causing occlusion of the gastric outlet. Ulceration was much less common in piroxicam-exposed WT mice (4 of 13 mice, 31%; Figure 2B; p<0.01 vs. *Sash* mice) and, when ulcers were present, they were smaller and did not result in gastric outlet obstruction (Figure 1E; Figure 2A).

### Susceptibility to severe ulceration and gastric outlet obstruction following piroxicam-exposure is mast cell-dependent

**Figure 3:**
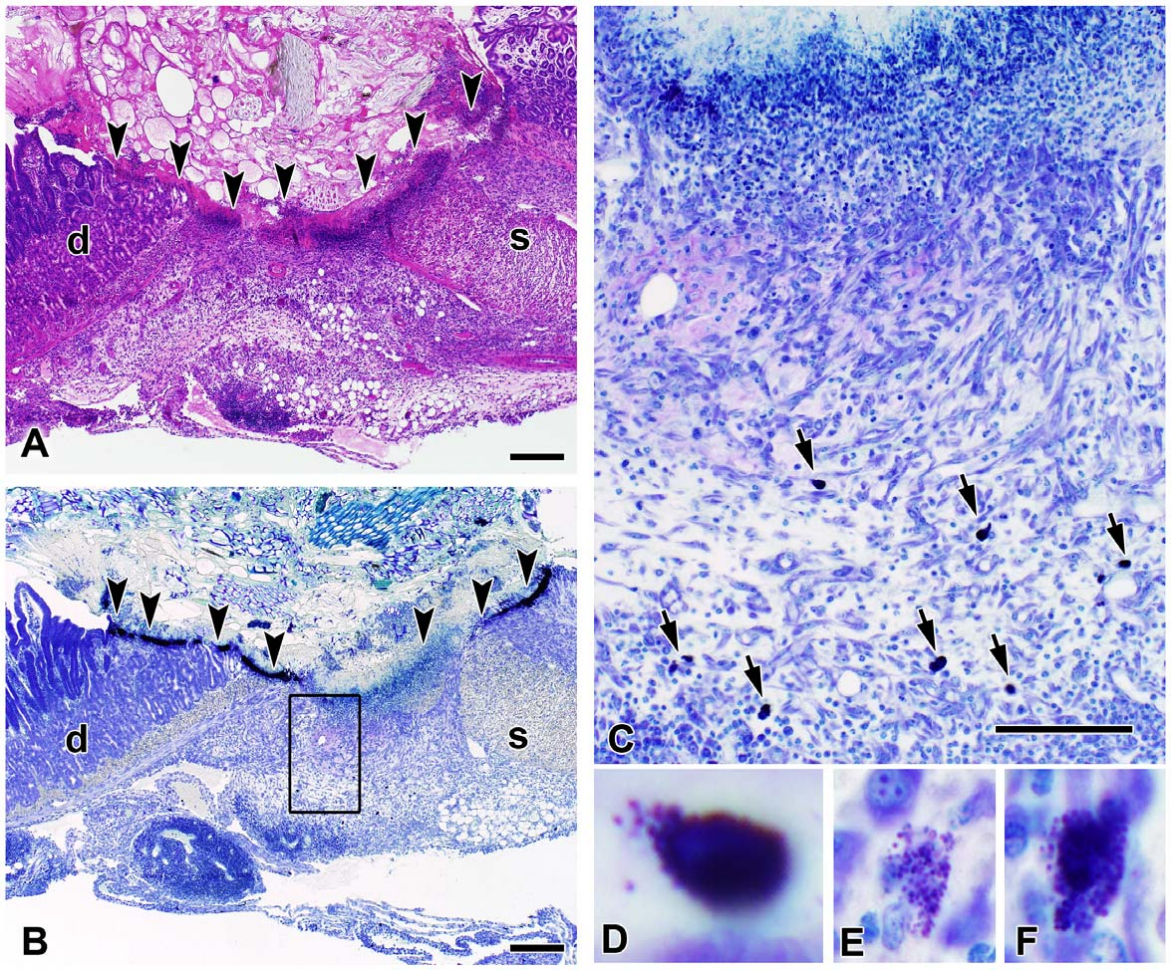
Mast cells in various stages of degranulation are present at the site of peptic ulceration. Hematoxylin and eosin- (panel A) and toluidine blue-stained sections (panels B–F) show a peptic ulcer located at the junction between the stomach (s) and duodenum (d). Arrowheads denote the extent of the ulcerated region. The intense purple staining adjacent to the arrowheads in panel B is derived from necrotic debris, inflammatory cells, and large numbers of metachromatic granules. Panel C shows the boxed region from panel B at higher magnification. Arrows point out mast cells in various stages of degranulation, as shown in panels D, E, F. Bar represents 200 µm in panels A and B and 100 µm in panel C.

To determine the role of mast cells in susceptibility to piroxicam-induced peptic ulceration, mast cell-deficient *Sash* mice [20], [21] were reconstituted with bone marrow-derived mast cells (BMMC) prior to piroxicam exposure. Reconstitution with BMMC eliminated the susceptibility of *Sash* mice to gastric outlet obstruction (Figure 2A) and restored the frequency of gastric ulceration in response to piroxicam to a level statistically similar to what is observed in WT mice (Figure 2B). Mast cells in various stages of degranulation could be observed adjacent to areas of ulceration at the gastrointestinal junction of both wild type and mast cell-reconstituted *Sash* mice (Figure 3).

### The protective effect of mast cells is not due to TNF production

Activated mast cells release granules containing histamine, heparin, basic proteinases (e.g. tryptase), and chemokines, as well as the pro-inflammatory cytokine TNF. Mast cell-derived TNF was previously shown to be critical for protection against infections and for development of adaptive immune responses against pathogens [22]–[24]. To test the role of TNF in NSAID-induced ulceration, mice globally deficient in TNF were exposed to 200 ppm piroxicam. Peptic ulcers occurred in 4 of 11 *Tnf* ^−/−^ mice and gastric outlet obstruction was observed in 1 of 11 mice. These incidence rates are statistically similar to what was observed in WT mice (Figure 2). Thus, TNF is not required for protection against peptic ulceration induced by piroxicam.

### Mast cell-derived histamine protects against piroxicam-induced peptic ulceration

**Figure 4:**
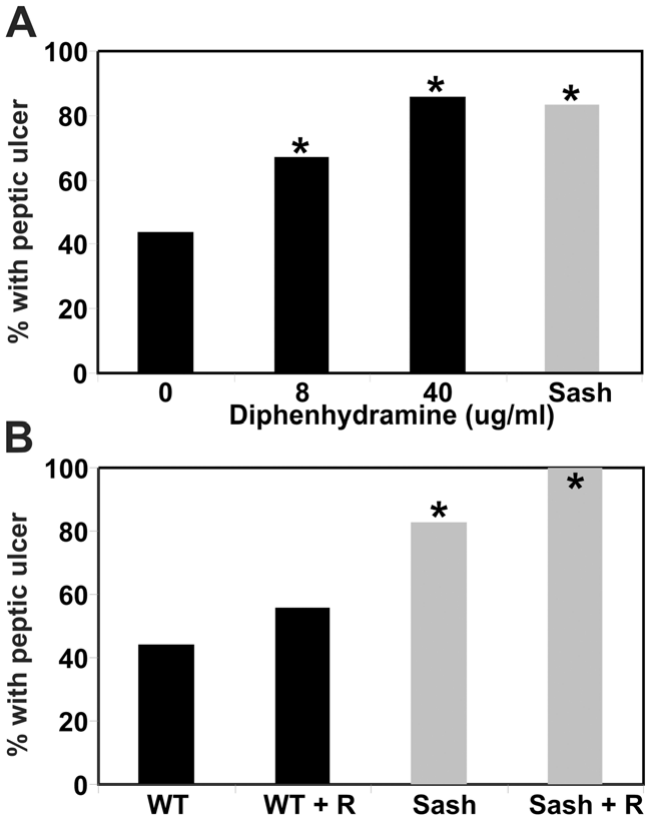
Effect of antihistamines on piroxicam-induced peptic ulceration. A. Treatment with the H1 receptor antagonist diphenhydramine increased histologically detected peptic ulcers in WT mice exposed to piroxicam (black bars; n = 7–9 per group), to a level comparable in mast cell-deficient *Sash* mice treated with piroxicam alone (gray bar; n = 18). Diphenhydramine did not affect ulcer incidence in *Sash* mice exposed to piroxicam (nor shown; n = 6; p<0.50). B. Treatment with the H2 receptor antagonist ranitidine (R; dose = 1.8 mg/ml in drinking water) did not affect the incidence of piroxicam-induced peptic ulcer in either WT (black bars) or *Sash* mice (gray bars). The numbers of mice studied were: WT (n = 13); WT+R (n = 9); *Sash* (n = 18); *Sash*+R (n = 7). For both panels, * indicates p<0.05 compared to WT mice treated with piroxicam alone.

To test the importance of histamine in piroxicam-induced peptic ulceration, WT and *Sash* mice were pre-treated with antihistamines provided in drinking water for 2 days prior to and during a 5 day exposure to piroxicam. Treatment with the histamine H1-receptor antagonist diphenhydramine hydrochloride did not affect the incidence or severity of peptic ulcers in piroxicam-exposed *Sash* mice (Figure 4), consistent with their lack of mast cell sources of histamine. However, similar treatment of piroxicam-exposed WT mice gave a dose-dependent increase in ulcers, with ulcer incidence at 40 µg/ml diphenhydramine equivalent to that seen in *Sash* mice (Figure 4A). In a separate study, treatment of WT mice with the histamine H1-receptor antagonist hydroxyzine hydrochloride at 40 µg/ml in drinking water prior to and during piroxicam exposure also showed a trend toward increased ulcer incidence (60%; n = 10). These data strongly implicate mast cell-derived histamine acting through H1 receptors as a mechanism that protects against NSAID-induced peptic ulceration. However, ulcers in antihistamine-treated WT mice were less severe than seen in mast cell-deficient *Sash* mice, both histologically and based on the lack of gastric outlet obstruction in diphenhydramine-treated WT mice. Treatment with the histamine H2-receptor antagonist ranitidine had no effect on the incidence of peptic ulcers in piroxicam-treated WT or *Sash* mice (Figure 4B).

### Inhibition of acid secretion does not protect against piroxicam-induced peptic ulcers

**Figure 5:**
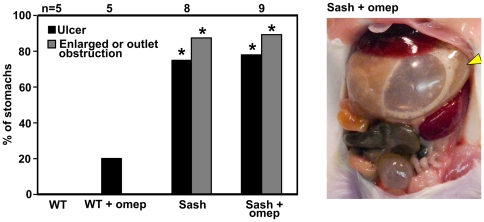
Acid inhibition with the proton pump inhibitor omeprazole does not protect mast cell-deficient mice from development of NSAID-induced peptic ulceration or gastric outlet obstruction. * indicates p<0.05 compared with WT mice treated with piroxicam alone. Gastric distension by large amounts of gas is readily apparent (arrowhead) in this gross photograph of an omeprazole-reated *Sash* mouse on day 5 of piroxicam treatment.

Blocking secretion of mucosa-damaging acid has been recommended to decrease the chance of NSAID-induced GI toxicity [25]. To test this, *Sash* and WT mice were treated with the proton pump inhibitor (PPI) omeprazole for 7 days prior to and continuing during the 5 days of piroxicam exposure. The pH of gastric lavage fluid in a separate cohort of mice analyzed after 7 days of PPI treatment increased from 2.9±0.3 (mean ± SEM; n = 8) in control mice to 4.4±0.3 (n = 3) in PPI-treated mice (p = 0.01). This corresponds to a decrease in acid secretion of 97%. However, despite this efficacy in acid reduction, omeprazole treatment did not decrease the incidence of piroxicam-induced peptic ulcers in *Sash* mice and most of these mice still developed marked gastric enlargement or gastric outlet obstruction (Figure 5). All mice with stomach enlargement >4× normal had ulcers at the gastro-duodenal junction. However, gastric enlargement 2–4× normal was seen in 2 *Sash* mice that had acute gastritis and edema of the stomach wall without frank ulceration. PPI treatment also did not affect incidence of piroxicam-induced ulcer in WT mice (1 of 5 mice = 20%; compare to Figures 1, 2).

### COX inhibition alone is insufficient to explain ulcer susceptibility in *Sash* mice

Piroxicam has significant activity against both COX-1 and COX-2 enzymes. To test the role of COX inhibition in ulcer susceptibility, *Sash* and WT mice were exposed to either the COX-1 inhibitor ketorolac, the COX-2 inhibitor etodolac, or both, formulated at 20 mg/kg powdered rodent chow for 7 days. None of these mice experienced significant weight loss or gastric enlargement suggestive of gastric outlet obstruction. Ulcers were seen histologically on day 7 in 1 of 5 *Sash* mice exposed to ketorolac, 0 of 5 mice given etodolac, and 0 of 5 mice given both drugs. Thus inhibition of COX-1, COX-2, or both enzymes alone seems inadequate to explain the gastric toxicity of piroxicam in *Sash* mice.

### Piroxicam impairs survival and proliferation of gastric epithelial cells

**Figure 6:**
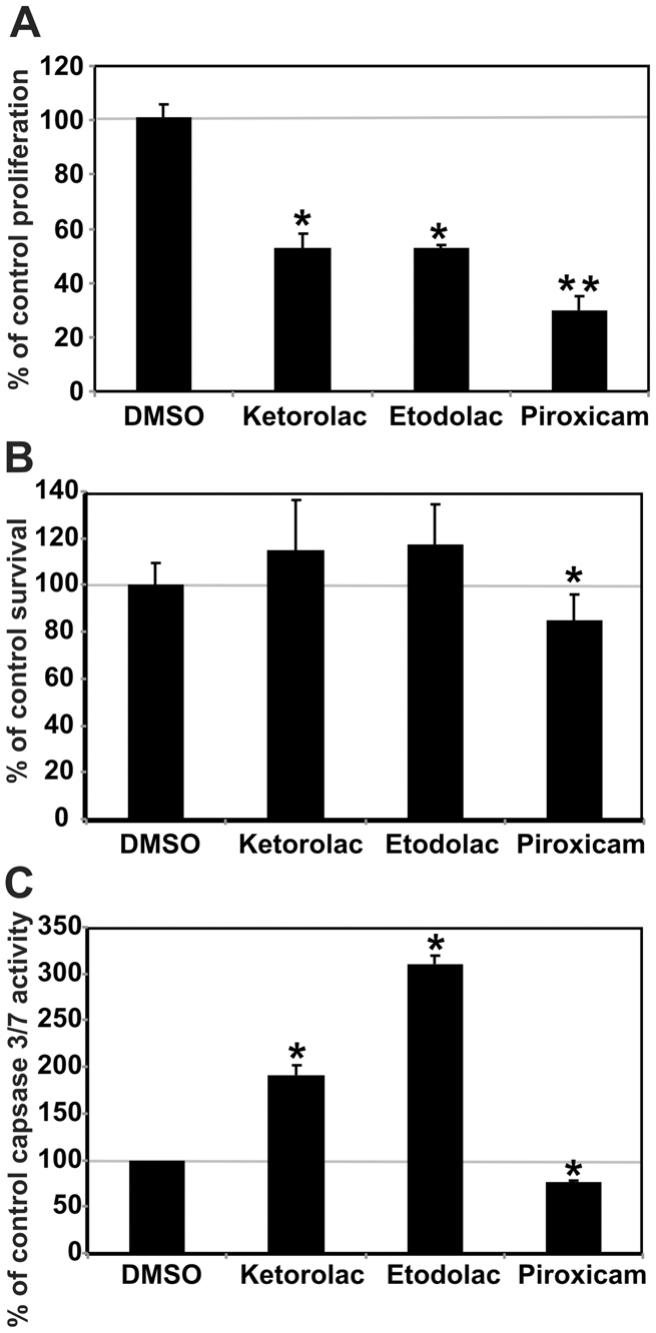
NSAIDs differentially affect apoptosis and proliferation of gastric epithelial cells in vitro. A. All three NSAIDs tested decreased proliferation of AGS cells compared with vehicle (DMSO) alone, as measured by changes in CFSE incorporation over 3 days of culture, with the largest decrease seen in piroxicam-treated cells (* indicates p<0.05 vs. vehicle; ** p≤0.04 vs. vehicle, ketorolac, or etodolac). B. Survival of AGS cells measured by a tetrazolium dye-based assay is significantly decreased by exposure to piroxicam, but not to keterolac or etodolac. Data shown (mean ± SD; n = 12) was obtained after 4 hrs of treatment. * indicates p<0.05 compared with DMSO control. C. Treatment with the NSAIDs ketorolac or etodolac, but not piroxicam, significantly increased apoptosis as indicated by increased caspase 3 and 7 activity, shown here at 24 hrs (mean ± SD; n = 3).

The AGS gastric epithelial cell line was used to further investigate the toxicity of NSAIDs on gastric epithelial cells *in vitro*. Ketorolac, etodolac, and piroxicam all reduced cell proliferation, but the magnitude of effect was greatest for piroxicam (Figure 6A). Piroxicam also reduced cell survival relative to control and to ketorolac or etodolac (Figure 6B). We previously showed that piroxicam induced apoptosis in colonic epithelial cells *in vivo* (40), however the decreased survival of piroxicam-exposed AGS gastric cells was not due to increased apoptosis (Figure 6C). Taken together, the data suggest that piroxicam has a direct toxic effect on gastric epithelial cells that both decreases their proliferation and decreases their survival via non-apoptotic mechanisms.

## Discussion

The data show that mast cell-deficient *Sash* mice develop peptic ulcers in response to oral exposure to piroxicam. These ulcers are severe, leading to occlusion of the gastric outlet by inflammation. Ulceration and gastric outlet obstruction in these mast cell-deficient mice are both prevented by restoring mast cells. This susceptibility to ulceration can be recapitulated by treating WT mice with diphenhydramine, an antihistamine that blocks the histamine H1 receptor. Taken together, these data indicate that mast cell-derived products, including histamine acting through H1 receptors, are vital for prevention and/or repair of NSAID-induced gastric epithelial injury.

The first line of gastric mucosal defense consists of luminal factors such as mucus, bicarbonate, and surfactants. Epithelial factors that provide further protection include tight adherence of cells, high resistance of apical membranes to acid, rapid extrusion of apoptotic cells, and rapid restitution following epithelial damage (e.g. a denuded rat stomach can cover 95% of its area with epithelial-appearing cells in 1 hour [26]). Extruded plasma can mix with mucus released by damaged epithelial cells to form a protective mucoid cap [27]. If these protective measures fail, inflammatory responses and ulcer healing are invoked [28]. Granulation tissue consisting of fibroblasts, macrophages, and angiogenic endothelial cells forms at the ulcer base within 48–72 hours. Viable epithelial cells at the ulcer margin de-differentiate, proliferate, and migrate onto the granulation tissue to cover the epithelial defect. Finally, these tissues undergo remodeling, leaving an underlying scar [29].

Involvement of mast cells in gastric ulceration and healing has been suggested previously [28], [30]–[34], but conclusive proof was not obtained for either effect or mechanism. In skin, mast cells primarily appear to be involved in the initial response to epithelial injury [reviewed in 35]. Mast cell degranulation occurs quickly in proximity to a wound [36]. Dermal wound healing is impaired in mast cell-deficient Kit^W^/Kit^W-v^ mice, with decreased immediate increase in vascular permeability and delayed wound closure [35], [37]. These findings could be reproduced by treating WT mice with a histamine H1-receptor antagonist, but not with H2-receptor or TNF antagonists. Scald wounds also showed delayed healing in mast cell-deficient mice [38].

We found a similar striking impairment of NSAID-induced gastric wound healing in mast cell-deficient *Sash* mice. Unlike the mast cell-deficient *Kit^W^/Kit^W-v^* mice used in previous studies [31], [39], *Sash* mice do not lack TCRγδ T cells and do not develop spontaneous gastric ulcers. However, they do appear to lack interstitial cells of Cajal, which may predispose them to bile reflux that can also be ulcerogenic [40]. The gastric outlet obstruction seen in NSAID-exposed *Sash* mice would completely eliminate any potential reflux of bile, although this does not rule out bile acid gastric toxicity as a component of the initial insult. It is also possible that the role of mast cells in reducing NSAID-induced injury may be different in WT vs. *Sash* mice. However, taken together, the observations that reconstitution of *Sash* mice with mast cells eliminated susceptibility to ulcers and that ulcer susceptibility could be reproduced in WT mice by blockade of histamine H1-receptors provides definitive evidence that mast cells are the primary factor in the observed phenotype, rather than epithelial abnormalities induced from bile reflux from the *Kit* ^W-sh^ genotype. Mast cell TNF production can also be eliminated as a mechanism for protection from gastric ulcers since TNF-deficient mice did not show increased ulcer susceptibility. Histamine can also be produced by platelets, but the similarity in phenotype between the *Sash* mice and wild-type mice treated with antihistamines suggests mast cell-derived histamine as the primary contributor.

Other mast cell-produced factors may also play a role in protection against NSAID-induced injury and/or facilitation of mucosal repair. FGF2 is produced by mast cells and can help prevent and heal NSAID-related ulcers [41], [42]. Higher mucosal tryptase levels were seen in humans infected with *H. pylori* strains that were less likely to cause ulcers, suggesting that mast cell-derived tryptase may promote mucosal turnover and healing [43]. Mast cell tryptase has also been shown to function as an epithelial mitogen following wounding [44].

It is important to note that these studies only used the NSAID piroxicam as an ulcerogenic stimulus. Thus additional studies will need to be performed to determine whether these results represent a NSAID class effect that can be exploited to limit the incidence of NSAID-induced side effects in humans. Despite considerable study, the physical and biochemical properties necessary for an NSAID to exhibit gastric toxicity remain poorly understood. The piroxicam concentration required to effectively inhibit COX enzymes *in vitro* is 1–5 µM [45]. Incubation with piroxicam concentrations ranging up to 100 µM inhibited release of histamine from rat peritoneal mast cells by a maximum of 25–34% [45], [46]. However, piroxicam at concentrations ranging from 0.001–100 µM did not affect IgE-induced histamine release from human synovial mast cells [47]. Since decreased histamine activity induced by administration of antihistamines that block H1 receptors makes wild type mice more susceptible to piroxicam-induced peptic ulceration, it is possible that decreased histamine release by mast cells in the presence of piroxicam may contribute to the ulcerogenic properties of this NSAID. However, inhibition of mast cell histamine release cannot account for the increased ulcer susceptibility observed in *Sash* mice, which lack mast cells.

Although the observations reported here were made in genetically-modified mice that lack mast cells, they have considerable relevance to human health. The recognition that mast cells and the histamine that they produce is important to mucosa during NSAID administration provides mechanisms that may be targetable to prevent NSAID-related gastric toxicity. Furthermore, although no mast cell-deficient humans have yet been reported, self-treatment with antihistamines at doses sufficient to impair mast cell function is common in atopic individuals. Concomitant NSAID and antihistamine use (e.g. in over-the-counter cold medications) also commonly occurs in the setting of common viral or bacterial respiratory infections.

The concept that histamine acting via histamine H-1 receptors may be necessary for appropriate ulcer healing may sound counter-intuitive, given that histamine H-2-receptor antagonists (H2RAs) are prescribed for acid reduction that is widely recognized to facilitate healing of peptic ulcers. Note that the H2RA ranitidine had no effect on ulcer incidence in our studies. A meta-analysis of trials concluded that standard-dose H2RA can prevent NSAID-induced ulcers in the duodenum of humans, but not the stomach [48]. Individual trials have reached similar conclusions with H2RAs either being ineffective or less effective at preventing gastric ulcers associated with aspirin or NSAIDs, either alone or in comparison to duodenal ulcers [49]–[53]. In any case, our studies clearly show that acid reduction cannot compensate for the lack of mast cells in protection against NSAID-induced gastric ulceration.

Taken together, our studies show that mast cells and histamine acting via H1 receptors play a heretofore unrecognized role in protection against and/or repair of ulceration induced by the NSAID-piroxicam. These findings corroborate known clinical information regarding diminished efficacy of H2RA in the prevention of NSAID-induced gastric ulcers and call for further investigation into the roles of mast cell-produced factors in the pathogenesis and repair of peptic ulcers induced by piroxicam or other NSAIDs.

## Materials and Methods

### Animal studies

Wild type C57BL/6 mice, mast cell-deficient *Kit^W-sh^/Kit^W-sh^* (*Sash*) mice (strain name = *B6.Cg-Kit^W-sh^/HNihrJaeBsmJ*; stock #005051), mice transgenic for global expression of green fluorescent protein (GFP) (strain name = *C57BL/6-Tg(UBC-GFP)30Scha/J*; stock # 004353), and mice deficient in TNF (strain name = *B6.129S6-Tnf^ tm1Gkl^/J*; stock # 005540) were obtained from Jackson Laboratories (Bar Harbor, ME). Where indicated, *Sash* mice were reconstituted by intraperitoneal injection of 10^7^ bone marrow-derived mast cells (BMMC) in 100 µl PBS. BMMC were derived *in vitro* as described previously [54] using bone marrow from GFP mice to facilitate documentation of reconstitution. Mast cell-reconstituted *Sash* mice were used 12–16 weeks post-reconstitution and compared with age-matched controls.

All animal studies were approved by the Institutional Animal Care and Use Committee of Duke University (protocol numbers A151-09-05 and A225-06-07). Sentinel mice exposed repetitively to dirty bedding from the mice used in this study were negative for parasites by microscopic exam, negative for *Citrobacter rodentium* by fecal culture, negative by serology for a panel of 22 murine protozoal, bacterial, and viral pathogens, including murine parvovirus, murine hepatitis virus, and murine norovirus, and negative for helicobacter species by fecal PCR.

### Model of NSAID-Induced Peptic Ulcers

NSAID was given in powdered rodent chow (LabDiet 5001, Purina, Framingham, MA) at 200 mg/kg diet for piroxicam and 20 mg/kg diet for ketorolac and etodolac. This dose of piroxicam was chosen since it has been commonly used in mice, particularly to trigger the development of chronic colitis in mouse strains that are genetically susceptible to developing inflammatory bowel disease (IBD), but does not cause colitis in WT or *Sash* mice [54]–[56]. Mice were monitored daily for weight loss and for food consumption on a per cage basis. Where indicated, omeprazole was added to powdered diet at 400 mg/kg diet, a dose previously documented to decrease acid secretion in mice [57]. The effect of omeprazole on gastric pH was confirmed following euthanasia by lavage of stomach for 1 min with 0.5 ml of purified water (pH 5.0). Collected lavage fluid was centrifuged for 5 min (14,000 rpm in a microfuge) prior to pH determination. Histamine antagonists were supplied in drinking water at 0.008 or 0.040 mg/ml for diphenhydramine or hydroxyzine hydrochloride and 1.8 mg/ml for ranitidine. Food and water consumption were measured to ensure comparable drug doses between groups.

Euthanasia was performed when mice lost >15% of body weight, became moribund, or had completed 5–7 days of NSAID treatment. After euthanasia, the stomach and duodenum were excised *en bloc* for examination using hematoxylin and eosin-stained paraffin sections. The primary outcome measure was the presence of ulcer in the stomach or duodenum. Granulation tissue in the stomach or duodenal wall was required for the diagnosis of ulcer. Lesser degrees of damage were classified as gastritis/enteritis (presence of neutrophils) or erosions (loss of epithelium). Gastric enlargement due to outlet obstruction was used as an indicator of ulcer severity and was classified grossly into categories of not enlarged, enlarged 2–4× normal, and enlarged >4× normal.

### In Vitro Studies

AGS gastric epithelial cells were obtained from the American Type Culture Collection (Manassas, VA) and cultured in F-12 Kaighn's nutrient mixture (Invitrogen Gibco 21127) +10% FBS+pen/strep with and without 1 mM piroxicam, etodolac, or ketorolac. Cell survival was assessed using a tetrazolium dye-based assay (CellTiter96 AQ, Promega). Relative amounts of apoptosis were determined by measuring activation of caspase-3/7 using a fluorescent substrate assay (Apo-ONE, Promega). Cellular proliferation was measured by flow cytometric quantitation of changes in content of carboxyfluorescein succinimidyl ester (Guava CellGrowth Kit, Guava Technologies).

### Data Analysis

The incidence of ulceration or gastric outlet obstruction between groups was compared using Fisher's exact (chi squared) test. Weight loss, gastric pH, and results from *in vitro* studies were compared using Student's t-test. A value of *p*≤0.05 was considered to be significant.
